# Distal Radial Artery Access for Coronary and Peripheral Procedures: A Multicenter Experience

**DOI:** 10.3390/jcm10245974

**Published:** 2021-12-20

**Authors:** Alexandru Achim, Kornél Kákonyi, Zoltán Jambrik, Ferenc Nagy, Julia Tóth, Viktor Sasi, Péter Hausinger, Attila Nemes, Albert Varga, Olivier F. Bertrand, Zoltán Ruzsa

**Affiliations:** 1 2nd Department of Internal Medicine, Division of Invasive Cardiology, University of Szeged, 6720 Szeged, Hungary; dr.alex.achim@gmail.com (A.A.); kakonyikornelmano@hotmail.com (K.K.); jambrikz@hotmail.com (Z.J.); drnagytfer@hotmail.com (F.N.); sasiviktor@gmail.com (V.S.); phausinger@gmail.com (P.H.); nemes.attila@med.u-szeged.hu (A.N.); vargaa@in2nd.szote.u-szeged.hu (A.V.); 2 Medicala 1 Invasive Cardiology Department, University of Medicine and Pharmacy “Iuliu Hatieganu”, 400000 Cluj-Napoca, Romania; 3 Bács-Kiskun County Hospital, Teaching Hospital of the Szent-Györgyi Albert Medical University, 6720 Kecskemet, Hungary; tothjulia0@gmail.com; 4 Cardiology Department, Quebec Heart-Lung Institute, Laval University, Quebec, QC G1V 0A6, Canada; Olivier.Bertrand@fmed.ulaval.ca

**Keywords:** distal radial access, radial approach, anatomical snuffbox, distal radial artery, radial artery, vascular complications, vascular access learning curve

## Abstract

Introduction: Distal radial access (dRA) has recently gained global popularity as an alternative access route for vascular procedures. Among the benefits of dRA are the low risk of entry site bleeding complications, the low rate of radial artery occlusion, and improved patient and operator comfort. The aim of this large multicenter registry was to demonstrate the feasibility and safety of dRA in a wide variety of routine procedures in the catheterization laboratory, ranging from coronary angiography and percutaneous coronary intervention to peripheral procedures. Methods: The study comprised 1240 patients who underwent coronary angiography, PCI or noncoronary procedures through dRA in two Hungarian centers from January 2019 to April 2021. Baseline patient characteristics, number and duration of arterial punctures, procedural success rate, crossover rate, postoperative compression time, complications, hospitalization duration, and different learning curves were analyzed. Results: The average patient age was 66.4 years, with 66.8% of patients being male. The majority of patients (74.04%) underwent a coronary procedure, whereas 25.96% were involved in noncoronary interventions. dRA was successfully punctured in 97% of all patients, in all cases with ultrasound guidance. Access site crossover was performed in 2.58% of the patients, mainly via the contralateral dRA. After experiencing 150 cases, the dRA success rate plateaued at >96%. Our dedicated dRA step-by step protocol resulted in high open radial artery (RA) rates: distal and proximal RA pulses were palpable in 99.68% of all patients at hospital discharge. The rate of minor vascular complications was low (1.5%). A threshold of 50 cases was sufficient for already skilled radial operators to establish a reliable procedural method of dRA access. Conclusion: The implementation of distal radial artery access in the everyday routine of a catheterization laboratory for coronary and noncoronary interventions is feasible and safe with an acceptable learning curve.

## 1. Introduction

The site of arterial access for coronary angiography and intervention has been the focus of research for decades as it is the source of major complications, especially bleeding. Over time, radial access has become the default option for most of us in diagnostic and procedural vascular interventions, but more recently, interest in distal radial artery access (dRA) has grown rapidly. A large body of literature has emerged describing several advantages of this novel technique over the conventional radial one: minimal risk of hand ischemia due to preservation of blood flow in the forearm, faster hemostasis, reduction in nursing staff time, greater comfort for the patient and the operator, the sheath is naturally secured by the curvilinear trajectory and, finally, it preserves the proximal forearm’s radial segment for future interventions or arterial graft harvesting [1,2,3,4,5].

Despite the booming fervor, concern has been expressed regarding the rapid uptake of dRA in the absence of high-quality safety data, a phenomenon possibly fueled by social media [6]. On the other hand, many operators are skeptical towards implementing dRA as standard practice because they are expecting clear benefits. We therefore aimed to undertake a systematic protocol with the inclusion of all comers in our study and collect a solid cohort of at least 1000 patients in order to evaluate the real-world feasibility and safety of both right and left dRA as default access sites for routine coronary angiography and all percutaneous coronary (PCI) and noncoronary interventions. Moreover, the study should deliver an in-depth analysis of the dRA rationale and its future perspectives. The main study motto was the transition to dRA access as part of a contemporary, quality improvement project suitable for all patients arriving at our catheterization laboratories.

## 2. Methods

A prospective, observational registry of 1240 consecutive patients with access via dRA was created from our databases. We collected deidentified data of all patients in whom a puncture of the radial artery in the area of the snuffbox or distal dorsal via either arm had been attempted between January 2019 and April 2021 in two cardiovascular centers in Hungary (Invasive Cardiology Division, University of Szeged, Hospital and Cardiology Division, Invasive Cardiology, Bács-Kiskun County Hospital, Kecskemét, Hungary) in a standardized form. Ethical approval was obtained from the hospital committee. Each patient signed their informed consent. The presence of any (even weak) palpable pulse in both the wrist and the anatomical snuffbox was the only eligibility criterion for enrolment. Due to the increased confidence of the four operators, patients with unstable hemodynamic conditions were not excluded, although in shock cases, primary femoral access was chosen. 

### 2.1. Materials and Technique

The details of the puncture technique have been described elsewhere (1–4). Regarding sheath and catheter material and size, we used identic products for distal and proximal access. The sheath used most frequently was “Glidesheath Slender” 5-French (Terumo Corp, Tokyo, Japan). The maximum sheath size that was introduced by dRA was 8.5-French, in a case of concomitant aortic valve valvuloplasty. The protocol for spasm prevention and treatment follows uniform principles, flushing the sheath with 0.2–0.3 mg of NTG (Nitroglycerine) i.a. and escalating to morphine IV in the case of prolonged and increasing pain. Pre-angiography, the NTG plus 2000 IU of heparin was universally administered to all our patients. At the end of the procedure, the sheath was removed immediately from the dRA and hemostasis of the radial access was achieved via compression in all cases (120 min), using a combination of one StatSeal Disc (Biolife, LLC, Sarasota, FL, USA) with gauze compression. Most frequently, diagnostic and guiding catheters were used with a standard length of 100 cm. In rare cases, where aortic elongation or patient size required longer material, 110 cm catheters were used (“Launcher”, Medtronic Inc., Minneapolis, Minn., USA or “Radiofocus Optitorque”, Terumo Corp, Tokyo, Japan). Complete hemostasis was confirmed for all patients the next day in the angiography suite by both physical examination and Doppler Ultrasonography (USG). Distal radial artery patency was confirmed by Doppler USG and other complications were also assessed. Lumen permeability was recorded by Doppler USG immediately after the procedure, at 24 h and at discharge, according to the local protocols. Routine follow-up was also performed in each patient to evaluate distal radial artery patency at each outpatient visit.

Compared to other studies, patients were instructed to grasp their thumb under the other 4 fingers in order to bring the radial artery to the surface of the radial fossa. The operator, who had extensive experience (more than 100 radial procedures performed), stood on the right side of the patient and the puncture was carried out, Doppler USG guided in each case, with a vascular linear transducer (7.5 MHz) in a sterile plastic bag.

### 2.2. Study Outcome, Definitions, Patient’s Selection

Technical success was defined as the completion of the planned dRA procedure without changing the access site. As mentioned before, the most important **inclusion criterion** was the presence of a pulse in the snuffbox. Not all patients underwent radial artery ultrasound before the procedure in order to assess vessel size and patency, only during puncture. The choice of the site of dRA (left or right) was made at operating physician discretion and patient preference after detailed evaluation of the patient’s pulses, medical history, and clinical case characteristics. The preprocedural **exclusion criteria** were:

(1) Ultrasound evidence of arterial occlusion, severe calcification, and a lumen of less than 1 mm;
(2) Established cardiogenic shock;
(3) Raynaud’s disease in the medical history.

The main outcomes were:

(1) Technical success;
(2) Access site complications (determined at the end of the procedure and at 1 day);
(3) The rate of crossover to another puncture site.

To standardize data collection and build a useful database, the following procedural details and access parameters were entered:

(1) Baseline patient characteristics (age, gender, height, weight, cardiovascular risk factors);
(2) Time to find the artery by Doppler USG;
(3) The total number of puncture attempts;
(4) Total access time, cannulation time, and puncture time (in seconds);
(5) Total procedure time (including fluoroscopy time);
(6) Indication for intervention, sheath size, catheter size;
(7) Postoperative compression time, compression type;
(8) Pain score (0–5);
(9) Radiation dose, contrast amount;
(10) Hospitalization time;
(11) Postoperative complications (listed below);
(12) Ultrasound measurement of arrtery diameters: distal radial artery in the anatomical snuffbox and proximal radial artery (2–3 cm of the styloid);
(13) USG-measured radial artery peak systolic velocity (PSV) (cm/s) and distal radial PSV (cm/s) by USG.

Anatomical considerations were also noted: high take-off, tortuosity, spasm, occlusion, plaque formation, calcium, brachiocephalic trunk tortuosity, and brachiocephalic trunk calcinosis.

Immediate vascular complications, such as hematoma, pseudoaneurysm, arterial occlusion, ischemic injury of the hand, compartment syndrome, arteriovenous fistula, infection, or the need for vascular surgery repair, were evaluated upon hospital discharge. All patients were scheduled for a detailed clinical follow-up examination at 3, 6 and 12 months after the procedure, and all complications related to the access site (late events such as artery occlusion, hematoma, arterio-venous fistula, nerve or bone damage) were recorded during these follow-up visits. 

### 2.3. Statistical Analysis

The current database was created using the Microsoft Excel 2019 program. Statistical analysis was performed using SPSS v26.0 software (IBM Corp., Chicago, IL, USA). Continuous variables are expressed as the mean ± standard deviation or the median with interquartile range (Q1, Q3). Categorical variables are presented as the count (percentage). The patient groups were compared using either the Mann–Whitney U test or the Kruskal–Wallis test. The threshold of statistical significance was *p* < 0.05. 

Written informed consent was obtained from all patients, and the Institution’s Ethics Committee approved the study.

## 3. Results

### 3.1. Baseline Characteristics

The clinical characteristics of the study population are shown in Table 1. The mean age of the patients (829 males (66.8%)) was 66 ± 12 years. Hypertension, diabetes, dyslipidemia, and smoking were present in 92%, 37%, 83%, and 25%, respectively. Radial access was achieved using the left (ldRA) (*n* = 300, 24.19%) or right arm (rdRA) (*n* = 940, 75.8%). The operator was allowed to decide whether to attempt puncture at snuffbox (SB) or distal–dorsal site (DD) or both. Stable clinical presentation accounted for 50% of indications, and 6% of patients had a history of coronary artery bypass graft operation. In 72% of the patients, PCI was performed (*n* = 895). The crossover rate in patients with PCI was similar to that of those with coronary angio alone (1.16% vs. 1.42%).

### 3.2. Ultrasonography Data 

The mean diameter of the right radial distal artery was 2.30 ± 0.2 cm and the diameter of the right radial artery more proximally was 2.45 ± 0.6 cm, the distal segment being therefore 6.52% smaller. There was no significant difference between the two segment diameters (*p* = 0.99). The results of specific preoperative ultrasound measurements are shown in Table 2. 

### 3.3. Procedural Data 

Most of the cases were performed through the right radial artery (*n* = 1108, 89.35%). SB was predominant (*n* = 1046, 84.35%). Access was obtained within 1.26 ± 1.1 min. The general procedural characteristics are summarized in Table 3. Moreover, two of the three main outcomes of our study interest can be yielded from this collection of data. The **overall technical success** rate, which was described as a successful dRA sheath insertion, was achieved in 1208 (97.4%) patients. It was slightly higher at the right dRA than the left dRA (97.65% vs. 96.51%, respectively, *p* < 0.05). Of note, among the total patients, only 32 cases (2.58%) required **crossover** to another access site. The most common crossover was from initial (failed) rdRA to ldRA. In 66 patients (5.32%), the preprocedural USG revealed the total occlusion of the artery. In this situation, as a first attempt, the contralateral artery had been punctured. These cases were not considered crossover situations because the initial access site defined by USG did not change. The overall femoral access of all patients during this period (not just those included in the registry) was 4.4%—this means that dRA has managed to perform in almost all types of interventions.

Regarding equipment, a 5F sheath (Terumo IS, Tokyo, Japan) and 5F diagnostic and guiding catheter were used most frequently in this registry, followed by 6.5F sheathless guiding (Launcher, Medtronic, Minneapolis, MN, USA) (*n* = 64, 5.1%). We also performed a few cases with 7-in-6F slender sheaths (*n* = 55, 4.43%), used in CTO-PCI.

### 3.4. The Impact of the Learning Curve

**Figure 1:**
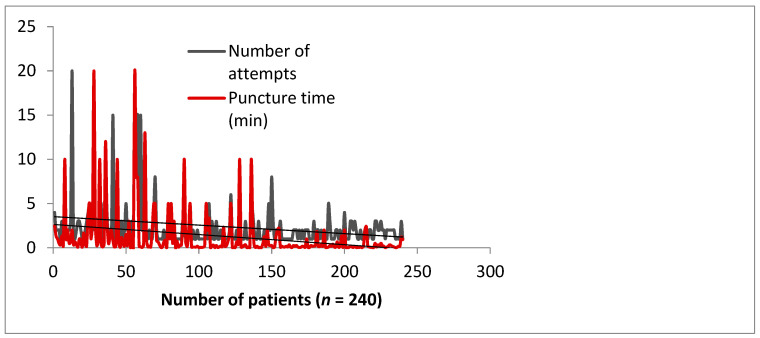
Learning curve impact on puncture time and number of attempts in 240 consecutive subjects, by 4 operators over a period of 3 months.

Although dRA access is a variation of traditional radial artery (RA) access, it has a distinct learning curve, mainly because the final segment of the RA moves in different directions along the carpal bones. Different types of analyses have been performed in an attempt to study the impact of the learning curve among our operators. A longitudinal analysis is illustrated in Figure 1.

Furthermore, we evaluated the performance index over a three-year period, namely (1) sheath time (composed of USG time, puncture to sheath insertion time, number of attempts, and penetration technique–anterior wall or transfixing puncture), (2) guiding cannulation time, and (3) total procedure time among a single operator who pioneered this technique in our center (Table 4). Throughout the years, there was no significant difference in procedure and catheter cannulation times, but the sheath parameters all improved, with a significant increase in ultrasonography scanning and accessing the artery speed.

We also tested the impact of the learning curve on a dRA-naïve operator (fellow) and, after experiencing 15 cases, the efficacy rate was already comparable with our main, the most experienced operator (Table 5). Of course, only the puncture time was kept almost double, to the detriment of the trainee.

### 3.5. Vascular Complications

The access site complication rate was acceptably low: at the end of the procedure, in 13 patients, there was some unfavorable alteration of the puncture area. No major bleeding occurred. However, some minor complications such as vasospasm (in six patients) and local hematomas (in four patients) were found. Morphine was used in only three cases of significant arterial spasm (0.2%). The most important safety issue, the occlusion of the radial artery, was observed in five patients (only 0.4% of the entire study population). However, the dissection of the artery wall without a significant compromise of the anterograde flow was found in five patients (0.4%) during the postprocedural USG. At 1 day follow up, no ischemic or further bleeding complications were observed. No patient complained about local numbness or any minor/major dysfunction of the hand, albeit four more (asymptomatic) radial artery occlusions were detected (0.32%). It means that the overall radial artery occlusion rate remained favorably low at 0.72%.

### 3.6. Noncoronary Interventions 

dRA was also attempted in 322 patients with endovascular treatment for noncoronary interventions. The endovascular procedures were focused on various cardiac and peripheral structures and clinical situations, as follows: balloon aortic valvuloplasty (3/322, 0.93%), internal carotid angioplasty (34/322, 10.65%), angioplasty for arteriovenous fistula dysfunction (3/322, 0.94%), vertebrobasilar insufficiency (11/322, 3.44%), thrombectomy for ischemic stroke (53/322, 16.61%), percutaneous superficial femoral artery interventions [7] (195/322, 61.12%), gastric artery embolization to treat severe obesity [8] (4/322, 1.25%), embolization for lung cancer bleeding (2/322, 0.62%) and one case of embolization for high-flow priapism (1/322, 0.31%).

## 4. Discussion 

In the current study, we assessed the technical feasibility of dRA in the endovascular treatment of various coronary interventions as well as noncoronary interventions such as BAV, secondary access for TAVI [5] and superficial femoral artery interventions [7], as some operators in our center have switched to dRA as common practice for all procedures since 2019. The dRA procedure demonstrated a high technical success rate and a low procedural complication rate that were comparable with conventional trans-radial access or transfemoral approaches. In addition, the mean time to achieve dRA was also comparable to that of conventional pRA (time to obtain arterial access of 1.26 ± 1.1 min) [9].

Complications were considered minor and low in incidence: hematoma development in 0.32% of cases, 0.4% arterial dissection, and 0.4% acute vessel occlusion but observing adequate flow after one month follow-up in half of them; no radial perforation, pseudoaneurysm, or arteriovenous fistula was observed. Ultrasonography revealed an important finding during the arterial access: wall dissection induced by the multiple needle and wire manipulations further led to wall micro-hematomas and acute vessel closure. This proved to be the reason for cannulation failure in most situations, with the impression of the operator that the vessel “has become spastic” being false. 

Our experience shows that the learning curve reaches an ideal mean of two puncture attempts performed under 30 s after 150 patients’ experience (Figure 1). Interestingly, Kim et al. reported a similar plateau success rate of 200 cases [10]. Performance indexes are still improving even after 2 years (Table 4), which means that mastering the dRA technique takes time, but it is an investment that will convince the operator to use it confidently in all types of interventions (acute, cardiogenic shock, aortic balloon valvuloplasty, CTOs, peripheral interventions, etc.). We consider that the use of ultrasound contributed to this performance, and it is highly recommended for a number of reasons. We have used ultrasound guidance in all cases because an anterior single-wall puncture can be carried out very safely with this technique, avoiding multiple punctures, and the site of the puncture can be selected in a nonangulated and nondiseased segment. The vessel is very superficial and is usually accompanied by one or two veins of similar size. Anatomical landmarks can be easily seen. Ultrasound has an advantage over a tactile location in that the operator can also measure the arterial diameter and determine whether the radial artery can accommodate the required procedural sheath and hardware, and choose a smaller diameter sheath in order to reduce the risk of vascular injury, unnecessary patient pain, and RAO. Flores E proposed a scheme for assessing the caliber of the artery in relation to risk for occlusion (artery diameter: high risk <1.8 mm, medium risk <2–1.8 mm, >2 low risk) [11]. Table 2 shows that the majority of our patients fell into the safe diameter category, with the cohort having a mean diameter of 2.3 ± 0.5 mm in the distal segment, easily accommodating a 5F or 6F sheath (the outer diameters of 5 French, 6 French, and 7 French introducer sheaths are usually 2.3, 2.6, and 2.8 mm, respectively) and no significant USG-measured caliber difference between the proximal radial artery and the distal radial artery was observed (*p* = 0.99). Lastly, blind puncture increases the risk of tendon and radial nerve damage. In current practice, many interventional cardiologists do not routinely use ultrasound, and so the additional requirement of adopting it in practice may contribute to the learning curve. Two meta-analyses [12,13] support the use of ultrasound guidance for conventional radial artery access vs. blind puncture with palpation as this leads to higher first pass success [12], quicker puncture time, and less hematoma formation [13].

**Figure 2:**
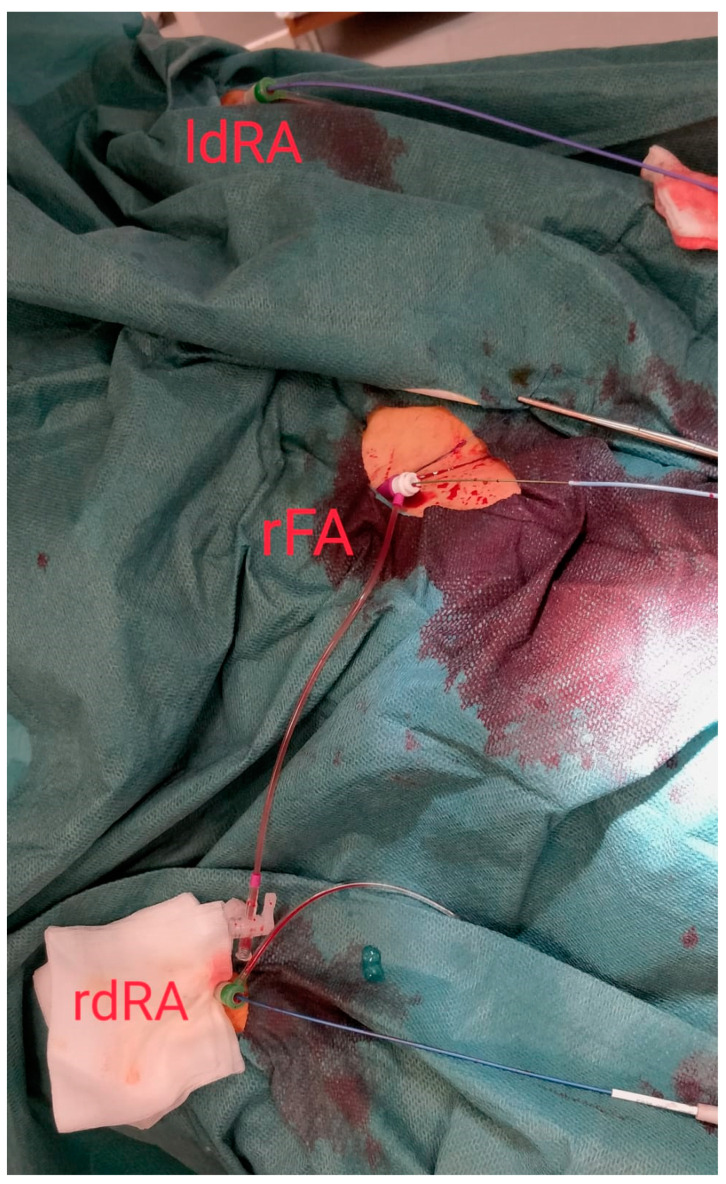
Increased ergonomics during TAVI: dual right (rdRA) and left (ldRA) distal radial access, with the main femoral access in the center (right femoral artery—rFA).

The artery segment being more distal, the diameter of the puncture site is obviously smaller, access is more difficult, and the learning curve is longer. In this study, the success rate of distal radial artery access was as high as 97%, the learning curve was acceptable, and the number of punctures was 1–3 times, therefore safety and efficacy are similar to the conventional approach. A common belief is that proficiency in dRA is difficult to gain and there is not enough data to support switching from conventional radial to dRA. Obviously, we may accept that the dRA will not fully replace standard transradial access as the default strategy, but it has a very high potential to improve the quality of access and intervention as a whole in many patients who prefer or require a procedure (especially via the left arm) in the presence of a palpable artery in the anatomical snuffbox. Long and arduous procedures such as CTO PCIs, which require dual access, choosing left or both dRA is one of the many examples where dRA has a clear benefit [14]. Figure 2 illustrates another useful situation, dual-dRA performed in routine TAVI, using both radial arteries as secondary access (one for aortography and one for periprocedural coronary protection), bringing the arms very close to the main femoral access, possibly lowering the radiation amount to the “femoral dose” and increasing workplace efficiency and comfort. The irradiation aspect is important and should not be forgotten as both hands are brought pronated over the patient’s pelvis, increasing the distance between the X-ray tube and the operator. 

Regarding the complication rate, we show similar numbers to all other studies published over the last 4 years [15,16,17]. It should be noted that, despite our study being one of the largest of all, complications remain infrequent. It is also clear from our observation that the technique can be rapidly mastered with the help of ultrasound and that the learning curve is no different from other manual procedures. dRA in combination with USG seems to eliminate the problem of radial artery occlusion at the forearm, together with better patient and operator comfort, a shorter compression time, and better outpatient PCI (3,4). 

While head-to-head comparisons between pRA and dRA have been started [18,19,20,21], we believe randomized, controlled trials directly comparing dRA to pRA are not sine qua non with regard to juxtaposition between the two sites as one does not substitute the other. dRA represents an evolution and a complement of the developing nature of TRA and nuances such as preservation of the common radial artery, easier hemostasis, and ergonomics play a role when choosing a specific entry site.

## 5. Conclusions

Our large cohort study with consecutive recruitment provides evidence that dRA is a reliable and safe vascular access site.

## Figures and Tables

**Table 1:** Baseline characteristics of all 1240 patients.

Demographic Features	Mean ± SD (Range)/*n* (%)
Age (years)	66.4 ± 12 (25–92)
Gender: female/male, % (*n*)	33.14% (411)/66.85% (829)
Height (cm)	173 ± 8
Weight (kg)	78 ± 17
**Risk Factors**	
Renal failure	196 (15.8%)
Diabetes Mellitus	463 (37.33%)
Hypertension	1152 (92.9%)
Smoking	311 (25.08%)
Family History	255 (20.5%)
Dyslipidemia	1035 (83.46%)
Previous MI	295 (23.79%)
Previous CABG	80 (6.45%)
Previous PCI	199 (16.04%)
**Indication for Catheterization**	
Stable Angina Pectoris	627 (50.56%)
Unstable Angina	60 (4.83%)
NSTEMI	82 (6.53%)
STEMI	46 (3.7%)
Heart Failure	16 (1.29%)
Severe Aortic Stenosis	25 (2.01%)
Cardiac Arrest	7 (0.56%)
Peripheral Interventions	322 (25.72%)

**Table 2:** Preoperative radial artery ultrasound measurements.

Parameters	Mean ± SD
Radial artery diameter, mm	2.45 ± 0.6
Distal radial artery diameter, mm	2.30 ± 0.5
Radial artery PSV (cm/s)	29.62 ± 10.5
Distal radial artery PSV (cm/s)	31.62 ± 17.2

**Table 3:** Procedural data.

Procedural Characteristics	Values
Coronary angiography only	272 (21.9%)
Coronary angiography and PCI	966 (77.9%)
Right distal transradial access	1108 (89.35%)
rdRA success rate	1082 (97.65%)
Left distal transradial access	172 (13.87%)
ldRA success rate	166 (96.51%)
Number of puncture attempts	2.28 ± 0.67
Artery puncture time, min	1.26 ± 1.1
Tortuosity (loop)	14 (5.9%)
Pain score (0–5)	2.7 ± 0.8
Sheath size (5F)	852 (68.7%)
Sheath size (6F)	269 (21.6%)
Sheath size (7F)	11 (0.88%)
Sheath size (8.5F)	4 (0.32%)
Procedural duration (min)	42.12 ± 10.1
Fluoroscopy time (min)	14.6 ± 10.2
Radiation dose (/mGy)	733.99 ± 542.23
Postoperative complications (total)	13 (1.04%)
Major bleeding	0 (0%)
Vasospasm	6 (0.48%)
Hematomas	4 (0.32%)
Artery occlusion	5(0.4%)
Hemostasis time (min)	225 ± 10
Repeat hemostasis	3 (0.24%)
Radial patency at discharge	4 (0.32%)

**Table 4:** One-operator performance index comparison (sheath time, cannulation time, procedure time) between 2019 and 2021.

	2019 (*n* = 550)	2020 (*n* = 448)	2021 (*n* = 242)	*p* Value
**Sheath Time**				
Ultrasonography Time (s)	23.9	20.5	11.82 ^c^	0.001
Puncture Time (s)	158	162	138 ^c^	0.001
Number of attempts	2.32	1.9	1.64 ^b^	0.02
Wall puncture Anterior Wall	261	223	148 ^c^	0.001
Anterior & Posterior Wall	202	214	92 ^c^	0.001
**Cannulation Time (s)**	16.65	15.3	15.9	0.14
**Procedure Time (min)**	38.1	45.2	41.48	0.13

Continuous data are presented as the median (interquartile range Q1, Q3); categorical data are given as the count (percentage). Significance level: ^b^ *p* < 0.05, ^c^ *p* < 0.01.

**Table 5:** The impact of the learning curve in a dRA-naïve operator.

Impact of the Learning Curve	Impact of the Learning Curve after 15 Cases	Control Group	*p* Value
Ultrasonography			
Finding time (s)	10.9 (5.2–16.7)	8.4 (4.8–12.2)	<0.0001
Radial cannulation			
Puncture time (s)	114.0 (58.8–19.2)	51.6 (40.7–62.6)	0.0358
Puncture attempts	2.15 (1.6–2.7)	1.6 (1.4–1.8)	0.0001
Procedural factors			
Procedure time (min)	41.3 (31.3–51.2)	33.2 (30.4–36.1)	0.0604
Contrast volume (mL)	106.4 (87.9–124.9)	108.7 (98.3–119)	0.8266
Radiation (Dyn/cm^2^)	457.1 (220–694.2)	472.2 (404.6–539.9)	0.0644

## Abbreviations

dRA	distal radial access
pRA	proximal radial access
RA	radial artery
USG	ultrasonography
IV	intravenous
NTG	nitroglycerine
RAO	radial artery occlusion
PCI	percutaneous coronary intervention
CTO	chronic total occlusion
BAV	balloon aortic valvuloplasty
TAVI	transcatheter aortic valve implantation
